# Report of Five Hundred and Seventy Cases Treated in the Military Hospital American Red Cross, Kiev, Russia

**Published:** 1915-11

**Authors:** Dean F. Winn

**Affiliations:** Atlanta, Ga.


### REPORT OF FIVE HUNDRED AND SEVENTY CASES TREATED IN THE MILITARY HOSPITAL AMERICAN RED CROSS KIEV, RUSSIA.

Dean F. Winn, M. D.,
Atlanta, Ga.

During the months between December 1st, 1914, and August 1st, 1915, the American Red Cross Hospital, located at Kiev, Russia, cared for more than four thousand wounded. Of these 570 came under my immediate care. Among these 570 individual there were:

| | | |
|---|---|---|
| Accidental injuries | 8 | |
| Acute appendicitis | 1 | |
| Acute adenitis (unknown origin) | 2 | |
| Tuberculosis adenitis | 1 | |
| Purely medical | 14 | |
| Frost bite | 28 | |
| Injuries account of concussion | 5 | |
| Wounded by bullets, etc. | 511 | |
| | | 570 |

Of the 511 cases of gun shot wounds, 108 suffered multiple wounds, making a total of 671 gun shot wounds. These injuries varied from slight lacerations to almost inconceivable mutilations and a composite picture of them would scarcely leave any portion of the body intact.

DISTRIBUTION OF WOUNDS:

| LOCATION | RIFLE BULLETS | | | | | | SHRAPNEL, Etc | | | | | | |
|---|---|---|---|---|---|---|---|---|---|---|---|---|---|
| | Perf. | | Pen. | | Loc. | | Perf. | | Pen. | | Lac. | | Total |
| | Inf. | Cln. | Inf. | Cln. | Inf. | Cln. | Inf. | Cln. | Inf. | Cln. | Inf. | Cln. | |
| Scalp | 1 | -- | -- | -- | 9 | 5 | 2 | 1 | 4 | -- | 15 | 2 | 35 |
| Cranium | 7 | 1 | -- | -- | -- | -- | -- | -- | 2 | -- | -- | -- | 10 |
| Face | 33 | 13 | -- | -- | 2 | -- | 8 | -- | 7 | 2 | 7 | 2 | 74 |
| Eyes | 4 | -- | -- | -- | 1 | -- | 1 | -- | 2 | -- | -- | -- | 8 |
| Ears | 1 | -- | -- | -- | 2 | 1 | -- | -- | -- | -- | -- | -- | 4 |
| Tongue | 9 | -- | -- | -- | -- | -- | -- | -- | -- | -- | -- | -- | 9 |
| Neck | 5 | 4 | -- | -- | 2 | -- | -- | -- | 1 | -- | -- | 1 | 13 |
| Pleura | 7 | 10 | -- | -- | -- | -- | 1 | 1 | -- | 3 | -- | -- | 22 |
| Thorax wall | 6 | 3 | -- | -- | 4 | 1 | 2 | -- | 6 | 1 | 7 | 1 | 31 |
| Abdomen | 7 | -- | -- | 1 | -- | -- | -- | -- | 1 | -- | -- | -- | 9 |
| Abd. wall | -- | -- | -- | 1 | -- | -- | -- | -- | 1 | -- | -- | 2 | 4 |
| Ex. genitals | 1 | -- | -- | -- | 3 | -- | 1 | -- | -- | -- | -- | -- | 5 |
| Shoulder | 11 | 14 | 2 | -- | 3 | -- | 4 | -- | 5 | 2 | 2 | 2 | 45 |
| Shoulder joint | 2 | -- | -- | -- | -- | -- | -- | -- | -- | -- | -- | -- | 2 |
| Axilla | 1 | -- | -- | -- | -- | -- | 1 | -- | -- | -- | -- | -- | 2 |
| Arm | 25 | 5 | -- | -- | 2 | -- | 10 | -- | 2 | -- | 2 | 1 | 47 |
| Elbow | -- | -- | -- | -- | 1 | -- | -- | -- | 2 | -- | -- | -- | 2 |
| Elbow joint | 3 | 1 | -- | -- | -- | -- | -- | -- | -- | 1 | -- | -- | 5 |
| Forearm | 18 | 6 | 1 | -- | 1 | 1 | 6 | -- | 5 | 1 | 4 | -- | 43 |
| Wrist | 6 | -- | -- | -- | -- | -- | -- | -- | 1 | -- | -- | -- | 7 |
| Hand | 12 | 10 | -- | -- | 1 | 1 | 4 | 1 | -- | -- | 3 | -- | 32 |
| Fingers | 18 | 3 | -- | -- | 3 | 1 | 7 | -- | -- | -- | 4 | -- | 36 |
| Lumbar reg. | -- | 1 | 1 | 1 | 2 | -- | -- | -- | 2 | 1 | -- | -- | 8 |
| Buttocks | 3 | 2 | 2 | -- | -- | 1 | 3 | -- | 10 | 1 | -- | -- | 22 |
| Hip | 1 | -- | -- | 2 | -- | -- | -- | -- | -- | -- | 2 | -- | 5 |
| Hip joint | -- | -- | -- | 2 | -- | -- | -- | -- | -- | -- | 2 | -- | 5 |
| Groin | 1 | -- | -- | -- | 1 | -- | -- | -- | 1 | -- | -- | -- | 3 |
| Thigh | 19 | 16 | 2 | 3 | 3 | -- | 9 | 2 | 10 | 6 | 1 | 3 | 73 |
| Knee | 1 | 3 | -- | 1 | -- | 2 | 2 | -- | 2 | -- | 4 | -- | 15 |
| Knee joint | 4 | 3 | -- | -- | -- | -- | 2 | -- | 1 | -- | -- | -- | 10 |
| Leg | 25 | 11 | 3 | -- | 3 | 1 | 13 | 1 | 2 | -- | 2 | -- | 61 |
| Ankle | 2 | 1 | -- | -- | 1 | 1 | -- | -- | 2 | -- | -- | -- | 7 |
| Ankle joint | -- | 1 | -- | -- | -- | -- | -- | -- | -- | -- | -- | -- | 1 |
| Foot | 9 | 1 | -- | 1 | -- | -- | 3 | 1 | 3 | -- | 2 | -- | 20 |
| Total | 242 | 109 | 11 | 9 | 42 | 13 | 80 | 7 | 73 | 17 | 55 | 13 | 671 |

There were 140 wounds of the head, 13 of the neck, 99 of the trunk, 221 of the upper extremity, 193 of the lower extremity and 5 of the external genitals. It will be seen that about 63 per cent. of the wounds were of the extremities and more in the upper than in the lower extremity. Probably the comparatively small number of trunk and skull wounds received was due to the fact that they are more frequently fatal.

Of the 140 head wounds, 10 injured the skull, 35 only the scalp and 95 were confined to the face. The chest was injured 53 times and the abdomen 13 times. 19 wounds of chest were perforating and 3 were penetrating. Of the wounds of the head 53% of those of the upper extremity 44.5 per cent. and of those of the lower extremity 30 per cent. caused fracture. Of the 123 lacerating wounds 70 were superficial. No bayonet or saber wounds were seen. . This seems strange since Russian cavalrymen carry swords and the infantrymen never remove their bayonets from their guns even in peace times. Further, both branches of the army are notorious for their zeal in hand-to-hand encounters. However, only one bayonet wound was seen in our hospital and that one was accidental.

Bullet wounds are by far the most frequent, there being in this series 426 wounds caused by bullets as compared with 245 caused by shrapnel, pieces of shell casing, etc. The accuracy of this information depended largely upon the statement of the soldier, but, checked up by the appearance of the wounds, it appears to be approximately correct. The general statistics of our hospital also show a predominance of bullet wounds.

Of the 371 bullet wounds (exclusive of lacerating) only 5.4 per cent. were penetrating; while of 177 shrapnel wounds (exclusive of lacerating) 50.6 per cent. .were penetrating. The mere lodgment of a missile in the tissues is, of course, not a matter of serious import unless it happens to be so situated as to impair the functions of the part or unless it acts as a source of infection. The chief claim of the above comparison to interest is that it reveals exactly what would be expected from the character of the missiles. Rifle bullets travel at great velocity and fail to perforate only when deflected by a bone—an infrequent occurence—or when striking after their force has been greatly diminished. The opposite holds true for shrapnel balls and casing fragments, and since they usually carry in with them particles of clothing a much larger percentage of infection follows—in this series 71 per cent. Of the penetrating bullet wounds only 55 per cent. were infected. Of the 110 total penetrating wounds there were 57 operations for removal of bullets, shrapnel balls, fragments of casing, etc. Clean penetrating missiles were not disturbed unless their presence was causing symptoms.

Of the 351 perforating bullet wounds 31 per cent. were clean, while of the 87 perforating shrapnel wounds only 8 per cent were clean.

Of 426 wounds by bullets, 30.7 per cent. were clean; of 245 wounds by shrapnel, etc.,. 14.5 per cent. were clean; of 671 total wounds, 24.8 per cent. were clean. We were not able to make microscopical examinations, but a low-grade, mixed infection was the variety usually encountered.

It is interesting to note that of the 505 infected cases there was no marked streptococcic infection except the five cases which developed erysipelas; only three cases had gas bacillus infection; and only three cases developed tetanus.

The large number of infected cases seen can hardly be attributed entirely to careless technic at the initial dressing. The Russian soldier is equipped with a sterile first-aid pack similar to that in use in the United States Army, and is taught how to use it. It is probable that the use of this pack does prevent secondary infection in many instances and its usefulness for this purpose should not be minimized. However, the majority of wounds are infected from the beginning owing to factors which are perhaps largely beyond control. In this war soldiers have had little opportunity for frequent baths and less opportunity for changing their clothing. Furthermore, the average Russian peasant, of which the army is chiefly composed, is constitutionally opposed to a bath except at long intervals and does not seem at all uncomfortable in soiled clothing. When one has seen the filthy uniforms and underclothing worn when they reach the hospital and the dirty skin beneath one marvels that there is ever a clean wound. And though the Russian soldier deserves his reputation for strength and endurance, there are few indeed who do not show evidence of the wear and tear of prolonged campaigning. This condition of lowered vitality, of course, encourages infection in his wounds.

Prophylactic vaccines are not used in the Russian army and I cannot say how this fact has affected our statistics. Answer to my inquiry about the non-use of these prophylactic doses was usually that they didn't do any good anyway. The real reason was probably that such products are not manufactured in Russia and that since the German market has been closed they have practically been dependent upon American goods. With only two ports open, one of which on the White Sea is frozen a good part of the year and the other six thousand miles away on the Pacific ocean, our own market was too far away to be of much value. This is only one instance of the dependence of Russia upon foreign countries for manufactured goods. Once, centuries ago, the Russian Princes sent out to Rurik of Sweeden this message:—"Our land is broad and our riches are many, but there is no order in our country. Come ye, therefore, and rule over us." Even at this late date there seems to be a perfect willingness to allow foreigners to reap the benefit from Russia's needs and resources.

Of the three cases of gas bacillus infection, one died. They were all marked by the peculiar odor, rapidly extending infection, extensive moist gangrene and intense prostration characteristic of this infection.

The five cases of erysipelas were evacuated, as soon as a diagnosis was made, to a special hospital for this class of cases. I was unable to procure information as to their mortality.

The three cases with tetanus were also promptly evacuated to the local Tetanus Hospital. One recovered and two died. No effort was made by us to treat them.

In case No. 68 symptoms first appeared 14 days after wound.

In case No. 310 symptoms first appeared 5 days after wound.

In case No. 408 symptoms first appeared 8 days after wound.

The majority of our tetanus cases were admitted after the snow began to melt and soil contamination became possible. Prophylactic doses of anti-tetanic serum, which have been so successful in checking tetanus in France, were not used, and I am informed that there was in Russia only a limited supply of serum in therapeutic form.

Thanks to a kind Providence no serious infection occurred amongst our personnel though we were compelled to work the greater part of the time without gloves and to literally wade in pus.

Of the 570 cases 12, or 2.1 per cent. died. The causes of death were as follows:

SURGICAL

| | |
|---|---|
| Septicemia | 6 |
| Edema glottis | 1 |
| Post-operative pneumonia | 1 |
| Traumatic Pneumonia | 2 |

(7 were operated on)

MEDICAL (not wounded)

| | |
|---|---|
| Advanced pul. tuberculosis | 1 |
| Tuberculous peritonitis | 1 |

Of the six dying of septicemia, one had an extensive, mutilating, compound, comminuted fracture of the humerus infected with gas bacillus. Amputation was done within a few hours after arrival six days after the wound occurred. One had a perforating wound of the popliteal artery and gangrene of the foot and leg. One had an extensive fracture of the neck and shaft of the femur. Another had a badly infected fracture of the upper femoral shaft, the infection extending from the iliac crest to the knee, and was moribund when received. One had a perforating, lacerating wound of the right thigh and a penetrating wound of the left knee joint which was badly infected. He was intensely septic. Another had a perforating infected wound of the knee joint.

One patient died from edema of the glottis. He had a badly comminuted fracture of the lower jaw with great destruction of the soft parts. . After removing loose pieces of bone and sloughing tissue an attempt was made to partially suture the wound. Embarrassed respiration followed the tying of the sutures and they were immediately cut. The patient could never be revived. Postmortem revealed no pathology to account for death other than acute edema of the glottis and larynx.

The death from post operative pneumonia followed an operation for compound fracture of the femur.

The patient dying from advanced pulmonary tuberculosis had no wound, and the death from tuberculous peritonitis occurred in a man whose wound through his knee joint had healed some time before admittance.

In two cases fatal traumatic pneumonia followed a perforating wound of the chest. In both instances the wounds had healed when admitted to the hospital.

Extreme exhaustation and intense toxemia played important roles in our hospital mortality which, in 4,000 cases, amounted to a little more than 3 per cent.

Of the eight accidental injuries there were 3 sprained ankles, 1 simple fracture of the radius, 2 simple fractures of the tibia, 1 simple fracture of the clavicle and 1 contusion of the right lower thorax.

The other surgical cases not wounded were acute appendicitis (1), acute suppurative adenitis of unknown origin (2), tuberculous cervical adenitis (1) and frost bite (28).

Of the 28 patients with frost bite, 13 were of 1st degree, 12 of 2nd degree and 3 of 3rd degree and were distributed as follows:—heel, toes, one foot, both feet, fingers, both hands, and ear. Having lived all my life in the South, the cases of frost bite were a matter of interest to me. Russian soldiers wear ill-fitting high leather boots and as a substitute for socks they wrap triangular cloths around the feet and ankles. In the majority of patients it was found that the boots were too small and the foot-cloths inclined to wrinkle, thus causing an embarrassed circulation. I have classified those cases with only swelling and reddening as 1st degree; those with sloughing of soft parts as 2nd degree; and those with gangrene of an entire member, for example, a toe, part of foot, etc., as 3rd degree. Frost bite was a large factor in permanently disabling soldiers and its prevention deserves attention from military authorities.

The following symptoms occurred in five individuals suffering from the effects of concussion only:—deaf mute, objective vertigo, vertigo and persistent headache, conjunctivitis and simple fracture of the radius and ulna. The last named attracted my attention because there has been some discussion as to whether the explosion alone of a shall could actually fracture a bone. In this case the forearm was black and blue throughout and the part fractured (middle third) bore no signs of having received any more trauma than other parts of the forearm. The soldier was quite sure that the shell did not strike him, but admits that he was knocked down by the force of the explosion. Another similar case was seen in the hospital.

Of the fourteen medical cases there were:

| | |
|---|---|
| Unilateral plastic pleurisy | 1 |
| Bilateral plastic pleurisy | 2 |
| Epididymitis | 1 |
| Chronic gonorrhoea | 1 |
| Chronic interstitial nephritis | 3 |
| Chronic Bronchitis | 1 |
| Advanced pulmonary tuberculosis | 2 |
| Acute indigestion | 1 |
| Carcinoma rectum | 1 |
| Syphilitic laryngitis | 1 |

Our hospital was purely for surgical cases and the small percentage of medical cases received were cases which accidentally passed the receiving station authorities. There was quite a good deal of cholera, typhus and typhoid in the section from which we drew, but they were handled entirely by other hospitals in Kiev devoted to those diseases. Medical cases were evacuated as promptly as possible because there was usually a pressing need in Kiev for space for the wounded, in spite of the fact that the city harbored some thirty thousand beds.

As a result of gun-shot wounds, the following complications were seen:

| | | | |
|---|---|---|---|
| Abdominal urinary fistula | 1 | Hypopyon | 1 |
| Adenitis, axillary (sup)) | 2 | Jaundice, septic | 1 |
| Adenitis, inguinal (sup) | 1 | Loss vision, both eyes | 4 |
| Aneurysm, femoral | 1 | Loss vision, one eye | 9 |
| Deafness | 5 | Neuritis | 3 |
| Decubitus | 5 | Paralysis, musc-spir | 1 |
| Deaf Mute | 1 | Paraplegia, partial | 1 |
| Empyema | 1 | Pneumonia, post-op. | 2 |
| Erysipelas | 5 | Pneumonia, traumatic | 3 |
| Gangrene | 8 | Septicemia | 5 |
| Gas bacillus infection | 3 | Stricture larynx | 1 |
| Hemiplegia, partial | 1 | Tetanus | 3 |
| Hernia, traumatic abdom. | 1 | | |

Other compliactions were:

| | | | |
|---|---|---|---|
| Acute tonsillitis | 1 | Organia heart lesion | 1 |
| Adenitis, cervical (t. b.) | 1 | Persistent vom. (neph) | 1 |
| Adenitis, cerv. & ax. (t. b.) | 2 | Peritonitis (t. b.) | 1 |
| Emaciation, extreme | 8 | Pleurisy, plastic | 2 |
| Epistaxis, profuse daily | 2 | Rheumatism, subacute | 8 |
| Hernia, ind. inguinal | 1 | Stricture rectum | 1 |
| Malaria | 1 | Tuberculosis, adv. pul. | 1 |

The abdominal urinary fistula followed an antero-posterior perforating bullet wound of the pelvis and healed promptly without surgical intervention.

In the case with traumatic femoral aneurysm the bullet passed tranversely through the lower third of both thighs, causing a clean fracture of the right femur and pinching a piece out of the left femoral artery. The fractured femur united with but little deformity. The aneurysm was operated on with good results. In a series of 671 gun shot wounds I was surprised to discover only one aneurysm. I have not the exact figures in hand, but the total number of this class of cases among the 4,000 wounded treated in the hospital was extremely small. However, we were not able to observe our patients for any great length of time since we were compelled to exacuate them as rapidly as possible. No doubt many of these patients will within the next twelve months develop false aneurysms.

The comparative absence of decubitus is a tribute to our nursing corps which was composed of twenty-five American and ten Russian nurses. It was no small tax upon them to efficiently care for 800 patients. The majority of our bed sores were present when the patients were admitted and were contracted in the crowded hospitals nearer the front.

The patient who developed empyema had a penetrating rifle bullet wound which entered in the fifth interspace, left mammary line, after perforating a St. George Cross which was suspended on the breast of his overcoat. The X-ray showed the bullet in the region of his appendix, having been deflected by the soldier's decoration for bravery.

The case with traumatic adbominal hernia had a perforating rifle bullet wound with the entrance at McBurney's point and exit above the right trochanter. The wound of exit was discharging pus freely. The wound of entrance was represented by a protruding sac about the size of an acorn with a granulating surface and containing omentum. There were no abdominal symptoms.

Traumatic pneumonia followed three gun shot wounds of the thorax and two deaths resulted. The case of post operative pneumonia followed a short operation for compound fracture of the femur. He was operated on promptly after being admitted and I do not know that pneumonia would not have developed without the operation.

One case of stricture of larynx resulted from a transverse bullet wound through the neck which mutilated the muscles beneath the tongue, fractured the inferior maxilla and hyoid bone and perforated the larynx. Deglutition was impossible and nasal tube feeding was necessary. In the beginning he had no trouble in breathing, but as the wound healed and the scar tissue contracted, respiration became more and more difficult so that two months after date of wound it became necessary to perform tracheotomy.

Physical examination of the chests of the majority of the patients revealed only one organic heart lesion. Likewise only one patient had a hernia.

Only two of my cases had dangerous hemorrhages as a late result of wounds. Both of these bled profusely from the base of the tongue and required suturing. Perforating wounds of the hard palate was a common cause of recurrent hemorrhage. Other causes of late hemorrhage were ulceration of large vessels by bone fragments and injuries to large vessels in the lungs. However, the relative number of dangerous hemorrhages was small.

In the beginning our attention was arrested by the enormous number of men with what we took to be Hutchinson's teeth. Closer investigation, however, revealed the fact that the notching was due to the universal habit of Russian peasants of eating sunflower seed which they crack open with their front teeth, and to the practice of biting off small pieces of lump sugar which they hold in the mouth while sipping tea. Russian recruiting officials inform me that no one suffering with gonorrhoea or spyhilis is admitted to the army. Judging from the few instances of these diseases discovered in our wards this rule must have been strictly adhered to. Both diseases are widely prevalent in Russia and inadequate treatment is the rule.

The following fractures occurred from gun shot wounds:

| | Infected | Clean | | Infected | Clean |
|---|---|---|---|---|---|
| Cranium | 9 | 1 | Ulna | 17 | 2 |
| One orbit | 5 | - | Carpal | 5 | 1 |
| Both orbits | 4 | - | Metacarpal | 11 | 1 |
| Ethmoid | 1 | - | Phalanges | 26 | 5 |
| Malar | - | 2 | Pelvis | 5 | - |
| Sup. Maxilla | 13 | 5 | Ribs | 1 | - |
| Inf. Maxilla | 33 | 1 | Femur | 15 | 5 |
| Nasal | - | 1 | Knee joint | 2 | - |
| Hyoid | 1 | - | Patella | 4 | 1 |
| Clavicle | 1 | 3 | Tibia | 12 | 7 |
| Vertebrae | 3 | 1 | Fibula | 6 | 4 |
| Scapula | 7 | - | Ankle joint | - | 1 |
| Humerus | 23 | 3 | Tatsal | 3 | - |
| Elbow joint | 3 | 1 | Metatarsal | 6 | - |
| Radius | 14 | 4 | | | |

Simple accidental fractures: Clavicle, 1; Radius, 2; Ulna, 1; Femur, 1; Tibia, 2.

Of 671 gun shot wounds 41.5 per cent. caused fracture.

Of 570 patients 43 per cent. had fractures.

17 per cent. of gun shot fractures were clean.

Of 221 gun shot wounds of upper extremity 44.5 per cent. caused fracture.

Of 193 gun shot wounds of lower extremity 30 per cent. caused fracture.

28 individuals had multiple fractures.

X-ray plates were made of most of the fractures of long bones. If the wound was clean, or if, though infected, there was not much comminution and the bullet wounds themselves were affording adequate drainage, attempt was made to correct the fracture by the use of Buck's extension apparatus, triangular splints for the humerus, and starch and plaster of Paris casts. 38 per cent. of the fractures of the long bones required operative interference. This consisted of free incisions, removal of bone fragments, bone dust and foreign bodies, and frequently resection of the proximal and distal fragments. The latter was accomplished with the aid of a Gigli saw without a great deal of difficulty. This was found to be necessary where the bone ends were denuded of periosteum and badly infected. No attempt was made to wire or plate the fragments except in two cases of fractured femur, one fractured humerus and one fractured ulna. These cases were wired largely as an experiment with full knowledge of the likelihood of failure. In the case of the femurs nothing was accomplished. The humerus united after draining for two months. At the time of discharge the patient had a tiny sinus which drained a small amount of thin pus, but there was good union of the bones. The wire will, of course, have to be removed, but I am inclined to believe he will have a useful arm. In the case of the ulna about two inches of both the radius and ulna were resected and extensive cellulitis was present in the forearm. I was particularly anxious to save his arm because he also had a fractured femur, and three fingers of the other hand missing. Not wishing to prolong the operation, only the ulna was wired. The wire was allowed to remain until the radius had united and was then removed. The infection cleared up after removing the wire and the ulna united satisfactorily. Supination and pronation are impaired by callus formation, but he has considerable use of his arm. This patient said that he was sitting with his left hand on his right forearm and with his right forearm resting on his right thigh, that while in this position a shrapnel shell struck him before bursting. The result was a badly mutilated left hand, compound fracture of ulna and radius and simple fracture of femur.

There were a few cases where the rifle bullet had bored a clean hole through the bone and thus caused little damage, but there were many more cases in which they had caused enormous destruction, failing to live up to their reputation as "humane bullets." Close range fighting, such as is taking place in this war, makes the rifle bullet an exceedingly dangerous missile to encounter. In the beginning we attempted to save many of the arms and legs where considerable comminution had occurred, but experience soon taught us that radical treatment—amputation—was sooner or later necessary and that it saved time, suffering and risk if done immediately. Our efforts with saving extremities was attended with much more success in fractured humeri than in fractures of the femur.

My experience leads me to believe that practically every high infected gunshot fracture of the femur should be amputated as soon as possible. This statement applies to wounds caused by high velocity bullets. Such radical action is probably not necessary in similar wounds eceived in civil war. With acutely infected compound fractures, even though loose fragments were known to be present, if the wounds themselves afforded efficient drainage, better results were obtained by waiting for some of the infection to subside than by hurrying into an operation.

In many cases where a good deal of bone had to be removed useful limbs were obtained by jamming the fragments together. When possible the fragments were wedged together in such a way as to assist in holding the bones in approximation.

Clean fractures with mal-union were commonly not disturbed on account of the risk of infection and the lack of the proper bone instruments.

Infected fractures involving the knee joint usually necessitated amputation.

Infected fractures of the elbow joint did well under the practice of resecting half of the joint. Excellent drainage was thus secured but ankylosis resulted.

Most of the fractures of the arm and leg were literally bathed in pus at the time they reached us. At the first dressing at the front plaster or starch casts were applied and the cast marked with the date of application and whether clean or infected. Frequently there was no mark to show locality of wounds. No windows were cut. Therefore, many of these men went from four to eight days without a dressing since that was the average time it took to transport them to Kiev. The result was that the original dressing corked up the wound, the pus burrowed about among the muscles and the patient got an extensive cellulitis and the full benefit of the accumulating toxins.

Three of the fractures of the skull had been operated on before reaching us. The remaining cases exhibited no pressure symptoms and were let alone. One of the cases which had been operated on developed intense headache and mental confusion but recovered promptly after small particles of bone had been picked out with tissue forceps. One patient had a small piece of shrapnel casing lodged in his brain 2 c. m. below a small wound of entrance in the left frontal region. He complained of no symptoms and the wound healed promptly without surgical interference.

Fractures of the ilium were all infected. On account of the spongy character of the bone, drainage to be adequate must be extensive. The prognosis is bad.

As a rule amputation flaps were not sutured. As the wound began to clean up the flaps were held together with adhesive straps. Surprisingly small scars were frequently obtained in this way. At times two or three stay sutures were inserted if the wound promised to be fairly clean. Amputation by transfixion was a common method used. Our catgut was not dependable so that large vessels were tied with silk. Muscle and skin flaps were rarely separated because the space thus made seemed to encourage the formation of pus pockets. Hip and shoulder joint amputations were done without the aid of transfixion pins.

Stimulation, good food, rest and careful nursing wrought remarkable changes in the wreck of humanity which filled our wards. Reams have been written within the past year with reference to the efficacy of old and new antiseptic dressings in gun shot wounds. I can readily understand the temptation to "try something new," for many of the low-grade, slowly healing wounds in patients whose resistance had reached a low ebb were quite discouraging. However, I saw no reason for departing from the rational procedure of multiple free incisions and tube drainage. Hot dressings of normal saline, potassium permanganate and bi-chloride of mercury were used when indicated. As a routine dressing, the use of Tr. Iodine and dry sterile gauze gave good results. Its use ought to be attended with common sense. Many patients were admitted with severe iodine burns as a result of a too free use of iodine at the initial dressing. Injection of 50 per cent. Tr. Iodine into sinuses hastened healing. When practicable, wounds were screened with wire netting and the part exposed daily to sunlight. This open method gave good results. Wounds were kept as dry as possible. Few cases were irrigated.

We were aided by the X-ray in locating bullets. The wound of entrance was no indication as to where the bullet had lodged and we discovered many freaky tricks they perpetrated. For instance, a bullet entered the outer side of the left shoulder, factured the left clavicle, then the right clavicle, passed through the right axilla and lodged in the right elbow. In another case four shrapnel balls were removed from the same hole in the heel which was the only wound the man had. The buttocks and thigh were the most common parts with retained bullets. Fragments of shell casing predominated with shrapnel balls a close second. Unidentified metal junk, pieces of clothing, leather, gun stock and wire were not infrequently found. Most of our patients came from the Austrian front and there is no doubt but that explosive bullets were used. Every soldier, if he had a mutilating wound, claimed it was caused by a dum dum bullet. In most instances these wounds were found to have been received in close range fighting and this fact probably accounted for their suspicious character. However, there were undoubtedly authentic instances of wounds from explosive bullets, and I have seen the unexploded cartridges taken from Austrian prisoners and also picked up on the battle field.

In all, 211 patients were operated on. 27 were done under cocaine anesthesia. The general anesthetics used were chloroform and ether. Chloroform was used more aften than ether on account of the shortage in the supply of the latter.

Sixty-one compound fractures required better drainage, removal of bone fragments, etc. The treatment was discouraging and constructive work practicably impossible on account of the ever present infection. By reason of the necessity for prompt evacuation, continued observation with reference to ultimate results could not be carried out.

There were 31 amputations including the arm, fingers, feet, forearm, leg, thigh and toes.

Ninety-eight operations were done for drainage of simple infected wounds, abscesses, removal of bullets, etc.

The following miscellaneous operations were performed:

Appendicitis, dissection of brachial plexus, dissection of tuberculous axillary glands, enucleation of eye, excision sebaceous cyst, orchidectomy, osteo-myelitis tibia, plastic on angle mouth, lower eye lid, upper and lower lip and tongue, repair traumatic abdominal hernia, resection femoral artery account aneurysm, suture tongue account hemorrhage, thoracotomy account empyema.

My experience in a military hospital would furnish abundant material were I inclined to add what is known as "sob sister stuff," but I believe the statistics given above need no comment in this direction on my part; they speak volumes for themselves.

Suite 714 Hurt Building.

